# Agricultural copper pesticide exposure and DNA methylation in Central Valley of California residents with and without Parkinson’s disease

**DOI:** 10.1016/j.envres.2025.122335

**Published:** 2025-07-11

**Authors:** Yufan Gong, Yu Yu, Keren Zhang, Irish Pearl Cambronero Del Rosario, Myles Cockburn, Laura K. Thompson, Adrienne M. Keener, Jeff Bronstein, Beate R. Ritz, Kimberly C. Paul

**Affiliations:** a Department of Epidemiology, Fielding School of Public Health, University of California, Los Angeles, CA, USA; b Center for Health Policy Research, University of California, Los Angeles, CA, USA; c Department of Population and Public Health Sciences, Keck School of Medicine, University of Southern California, CA, USA; d Department of Neurology, David Geffen School of Medicine, University of California, Los Angeles, CA, USA; e Department of Environmental Health Science, Fielding School of Public Health, University of California, Los Angeles, CA, USA

**Keywords:** Copper pesticide, DNA methylation, Parkinson’s disease

## Abstract

**Background:**

Copper-induced oxidative damage and mitochondrial dysfunction may exacerbate Parkinson’s disease (PD) pathology. DNA methylation (DNAm) can serve as a biological mediator of environmental exposures.

**Objective:**

To investigate the impact of chronic, long-term copper exposure on genome-wide DNA methylation patterns in the blood of PD cases and community controls.

**Methods:**

We analyzed 806 blood samples (569 PD cases and 237 controls) from a population-based case-control study. Epigenome-wide association analysis (EWAS) was conducted to identify differentially methylated probes (DMPs) and regions (DMRs), adjusting for age, sex, European ancestry, smoking status, study wave, cell composition, and organophosphates (OP) exposure. In addition, overrepresentation analyses were conducted to identify enriched pathways and biological functions.

**Results:**

We identified 140 CpG sites and 40 genomic regions that were associated with copper pesticide exposure, including cg17591573 (r = −0.16, p = 8.84E-09) in the *COL11A2* gene, cg11538176 in *UBE3C* (r = −0.18, p = 9.26E-09), cg11208322 in *MAGI2* (r = −0.15, p = 1.00E-08) and a region located on chromosome 1 (bp start site 205818668; ends 205819609). DMP and DMR gene set enrichment analyses identified as the most enriched pathways Fc gamma R-mediated phagocytosis (p = 6.03E-05, FDR = 2.17E-02) and homophilic cell adhesion via plasma membrane adhesion molecules (p = 2.27E-07, FDR = 5.08E-03).

**Conclusion:**

We found evidence that long-term ambient exposure to copper pesticides resulted in differential DNA methylation in blood of PD patients and elderly controls. Chronic copper pesticide exposure may affect multiple pathways, specifically phagocytosis pathway, EGF receptor signaling pathway, and homophilic cell adhesion mechanisms that are relevant to neuroinflammation and neurodegeneration in PD.

## 1. Introduction

Prolonged exposure to pesticides has consistently been linked to Parkinson’s disease (PD), the second most common neurodegenerative disease (Ohlander et al., 2022; Paul et al., 2023; Yan et al., 2018). PD is known for progressive loss of dopaminergic neurons, abnormal accumulation of *α*-synuclein protein (known as Lewy bodies) in the brain (Kouli et al., 2018), and also for inflammatory action in the central and peripheral nervous system (Pajares et al., 2020). It has been suggested that copper-induced oxidative damage and mitochondrial malfunction may facilitate these pathophysiological processes contributing to PD (Pyatha et al., 2022). While it is not yet understood how copper exhibits its toxicity in PD, its ability to directly interact with cysteine residues of proteins and strong redox activity may contribute to inactivation of enzymatic activity or even to neuronal death in the substantia nigra (Letelier et al., 2009; Carboni and Lingor, 2015; Bisaglia and Bubacco, 2020).

A recent animal study of juvenile male mice dosed with copper sulfate in drinking water for 40 weeks, found that copper exposure may contribute to dopaminergic neuronal loss, motor dysfunction, and α-synuclein accumulation and aggregation in vivo (Gonzalez-Alcocer et al., 2023). In our PD progression study, we recently found that high levels of ambient exposure to copper sulfate (pentahydrate) increased the risk of motor progression, cognitive decline, and depression with hazard ratios (HR) between 1.22 and 1.36 per standard deviation increase in exposure (Li et al., 2023). Our subsequent experimental study suggested that copper sulfate (basic and pentahydrate) treatment can lead to cell death in midbrain dopaminergic neurons (mDA) derived from PD patients’ induced pluripotent stem cells (iPSCs) (Paul et al., 2023).

DNA methylation (DNAm) may serve as a biological mediator in the pathways from environmental pesticide exposure to PD (Schaffner and Kobor, 2022), as it is known as a gene expression regulator that is sensitive to environmental exposures. For example, we previously identified 70 differentially methylated CpG sites in blood that were associated with organophosphate (OP) exposure among 579 study participants (342 PD cases and 237 controls) (Paul et al., 2018). However, few population-based epigenome-wide studies have investigated individuals exposed to copper pesticides, and our understanding of epigenetic changes due to copper exposure is limited. Here, we included the above-mentioned 579 participants and added 227 additional early-stage PD cases and relied on ambient copper exposure data from agricultural applications near homes and workplaces derived from the California Pesticide Use Reporting (CA-PUR) system to assess DNA methylation in blood.

Biologic pathways identified through the interrogation of epigenetic changes in blood cells of individuals exposed to copper-based pesticides may improve our understanding of how copper might elucidate chronic neurotoxicity related to PD risk or faster progression.

## 2. Method

### 2.1. Study population

We obtained data from the Parkinson’s Environment and Gene (PEG) Study that explores environmental risk factors contributing to PD in agricultural areas of the Central Valley of California. A total of 806 participants (569 PD cases and 237 controls) with DNA methylation profile are included in this study.

The methodology for data collection has been outlined in previous publications (Costello et al., 2009; Paul et al., 2015; Wang et al., 2011). In brief, the PEG Study comprises two distinct phases: PEG 1 (2000–2007) and PEG 2 (2010–2016). During PEG 1, recruitment involved collaboration with large medical groups, neurologists, and public service announcements, resulting in the enrollment of 342 eligible out of 1,167 screened patients who provided information for this study. In PEG 2, the pilot PD registry program in California was utilized to reach out to patients, and 227 eligible individuals, out of 2,713 patients from the PD registry we screened, provided all required information for this study. To meet eligibility criteria, PD cases had to fulfill the following conditions: 1) currently reside in Kern, Tulare, or Fresno County (tri-county area); 2) have lived in California for at least 5 years before PD onset; and 3) be in an early stage of PD (i.e., diagnosed with PD within 3 years of recruitment in PEG 1, extended to 5 years in PEG 2); 4) provide at least one blood sample. All eligible PD cases underwent examinations, often multiple times, by movement disorder specialists from the University of California, Los Angeles (UCLA), and were clinically considered to be idiopathic PD cases.

Population controls with comparable age and sex distributions to the patient population we enrolled were identified from Medicare lists in 2001 and from residential tax assessor records during the entire time PD patients were enrolled. To meet eligibility criteria in this study, controls had to: 1) be at least 35 years old; 2) reside in the tri-county areas where patients lived; 3) have lived in one of the counties for a minimum of 5 years before enrollment; and 4) have not received a diagnosis of parkinsonism; 5) have undergone at least one blood sample collection. Further details on eligibility criteria are available in our previous studies (Ritz et al., 2016). Population controls were enrolled through mail or phone contact. Out of 1,212 screened controls, 408 met all the inclusion criteria and were enrolled, and 237 of them provided blood samples and were included in the methylation array. All procedures outlined here received approval from the institutional review boards of UCLA, and all participants provided informed consent.

### 2.2. Pesticide exposure estimation

We employed a geographic information system (GIS)-based model to estimate ambient copper-related pesticide exposure proximity at both residential and workplace addresses, utilizing data from the California Agricultural Pesticide Use Reporting (CA-PUR) system which was mandated by law since 1974. To outline the exposure assessment process briefly: 1) participant lifetime address histories were collected, covering both residential and occupational history through interviews; 2) addresses were geocoded based on latitude and longitude information; 3) the geocoded location histories were recorded annually to match the annual CA-PUR data, incorporating land-use survey data, to generate annual measures of pounds per acre for each active ingredient applied within a 500m radius buffer around each address. This method resulted in person-year-location records that aligned with the annualized CA-PUR data.

In our study population, we identified residential or workplace proximity to the application of 15 specific chemicals classified as copper pesticides (Table 1). This determination was based on information obtained from the California Department of Pesticide Regulation (CADPR) and the Pesticide Action Network (PAN) pesticide database. We first calculated the average poundage of copper pesticides applied within the 500m buffer surrounding both participants’ residential and occupational addresses from 1974 to the year of the blood draw. Then we dichotomized the yearly average exposure of copper pesticides according to the median levels observed in healthy controls. Subsequently, we calculated the total number of copper pesticides to which each participant was exposed above the median value. This process resulted in a copper count ranging from 0 to 17, representing the measures of copper exposure.

OP pesticides have also historically been widely used in the Central Valley and linked to DNAm and PD. We observed strong Pearson correlation coefficients between copper and OP exposure of 0.76, 0.75, and 0.76 in the total study sample, among PD patients, and among non-PD controls, respectively (Table 1, Supplement Fig. 2–4). Thus, we applied the same methods described above to calculate OP counts to be able to adjust for potential confounding by OPs.

### 2.3. DNA extraction and Illumina methylation array

Peripheral whole blood was the source for DNA extraction in our study. The DNA samples underwent profiling using the Illumina Infinium 450k platform, encompassing 486,000 CpG sites. This array provides coverage for 99% of RefSeq genes, featuring an average of 17 CpG sites per gene region that spans the promoter, 5′UTR, first exon, gene body, and 3′UTR. Additionally, it includes 96% coverage of CpG islands, with additional representation in island shores and the surrounding regions (as per the Illumina Infinium HumanMethylation450 BeadChip data sheet) and the concordance of sex was verified. The raw methylation data, represented by beta values, underwent preprocessing using the background normalization method within the Genome Studio software. Then, cross-reactive probes, probes on SNPs, probes on sex chromosomes, and non-CpG probes were filtered out to avoid spurious associations caused by technical artifacts or unstable performance, which led to a total of 349,789 CpG sites left in the beta-matrix. Since extreme values within the matrix could greatly influence the epigenome-wide association analysis (EWAS) result, we further winsorized the betamatrix by each feature to 5th or 95th percentile (i.e., replacing the values above the top 5% or below the bottom 5% with the 95th percentile or 5th percentile values).

### 2.4. Statistical analysis

We first assessed the relationship between copper-related pesticide exposure and DNA methylation level by performing EWAS. A residual matrix was created using the empirical Bayes linear model in the *WGCNA* R-package to remove contributions from potential confounders [i.e., age at blood draw, sex (female and male), European ancestry (derived from genetic principal components), smoking status (self-reported as non-smokers, former smokers and current smokers), study wave (PEG1, PEG2), cell composition (i.e., CD8T, CD4T, NK, Bcell, neutrophil, granulocytes), and OP counts]. The cell type proportions were estimated based on DNA methylation levels and described in our previous publication (Horvath and Ritz, 2015). Specifically, the Housemen estimation method (Houseman et al., 2012) and the epigenetic clock software (Horvath, 2013; Horvath et al., 2015) were used. As shown in a DAG (Supplement Fig. 1), DNA methylation has been suggested as a mediator between pesticide exposure and PD, representing indirect effects of pesticides on PD (Schaffner and Kobor, 2022). Pesticide exposure may additionally also have a direct effect on PD development. Thus, we did not add PD status to our models to avoid inducing a collider (selection) bias. However, PD also contributes to DNA methylation changes and it influences immune responses reflected in methylation (Horvath and Ritz, 2015), such that DNA methylation can be both a mediator and a consequence of PD. To address this, we performed analyses in the total study population and subsequently also stratified by PD status.

We applied the *ChAMP* R-package to identify differentially methylated probes (DMP) and differentially methylated regions (DMR) by regressing the adjusted individual CpG methylation levels (residuals) on copper count measurements. Specifically, the empirical Bayes regressions from the *champ.DMP* function and the *Bumphunter* algorithm from *champ.DMR* function were utilized (Jaffe et al., 2012; Morris et al., 2013; Smyth, 2004; Wettenhall and Smyth, 2004). The obtained adjusted p-values were used to evaluate statistical significance and served as input for the overrepresentation analysis. We then calculated bi-weight mid-correlation coefficients between the adjusted beta values of each CpG site and the corresponding copper exposure to test for association. This helped summarize the strength and direction of association between adjusted methylation beta values and copper exposure. Genomic inflation factors (*λ*) for the EWAS models were also calculated. A high inflation factor can indicate potential issues, such as population stratification/confounding. Multiple testing (as there are ~350k CpGs in the Illumina array after filtering) was taken into account through Benjamini-Hochberg correction, resulting in an unadjusted p-value <10E–07 for a statistically significant association.

To gain insight into biological function of the differentially methylated CpG sites related to copper exposure identified through EWAS, we conducted a gene set overrepresentation analysis by applying the *gometh* method in the *ChAMP* and *missMethyl* R-packages to identify the enriched Gene Ontology (GO) terms and Kyoto Encyclopedia of Genes and Genomes (KEGG) pathways. This method uses the number of CpGs contained in genes to correct the p-value and to increase the reliability of results (Geeleher et al., 2013; Phipson et al., 2016; Young et al., 2010). In addition, we also utilized the *PANTHER* classification system and *methylGSA* R-package to identify overrepresented genetic pathways and functions for CpG sites found to be differentially methylated by mapping gene ENTREZIDs to PANTHER IDs (Muller, 2023; Ren and Kuan, 2019).

All data analyses were performed in R 4.3.3 (R Foundation for Statistical Computing, Vienna, Austria).

## 3. Results

### 3.1. Demographic characteristics

The mean age of our total study population of 806 subjects was 69.6 and patients were slightly older than controls (70.5 ± 9.8 vs. 67.4 ± 12.8) at the time of blood draw (Table 1). Most of the study participants were of European ancestry (n = 695, 86.2%) and more were male (n = 482, 59.8%) as PD is more common in men. A total of 409 individuals (50.7%) had a history of smoking but most were former smokers. On average, PD patients were exposed to more copper (3.9 ± 4.1 vs. 2.8 ± 3.3) and OP pesticides (18.2 ± 13.8 vs. 13.6 ± 11.7) than controls.

### 3.2. Epigenome wide methylation analysis

**Fig. 1.**
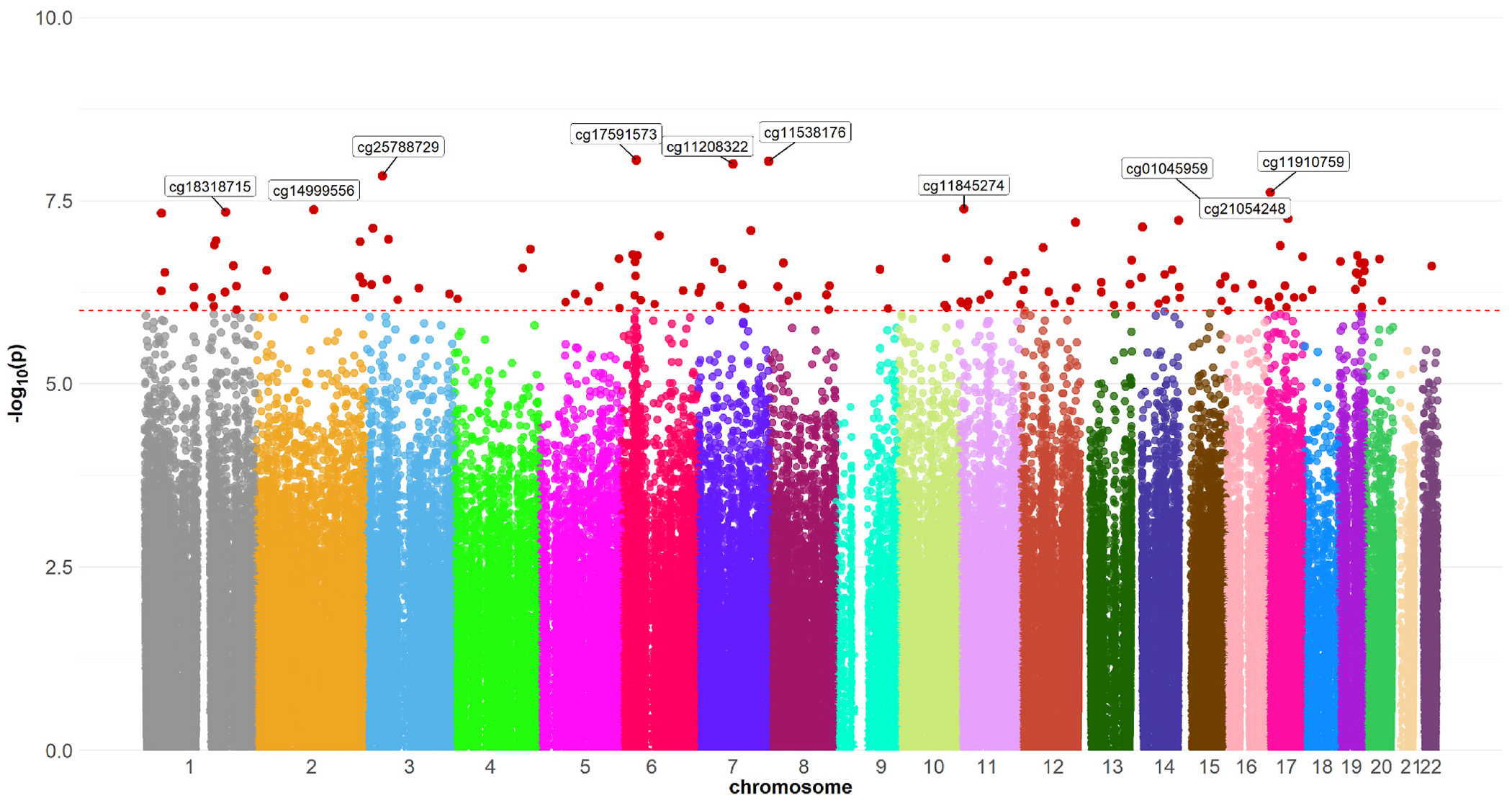
Manhattan plot for copper pesticide exposure and methylation in the total study population (n = 806). The red dashed line represents the threshold for statistical significance (-log10(p) = 6). A total of 140 CpG sites were identified as differentially methylated with copper exposure (above the red dashed line). The top 10 CpGs are labeled. (For interpretation of the references to colour in this figure legend, the reader is referred to the Web version of this article.)

**Fig. 2.**
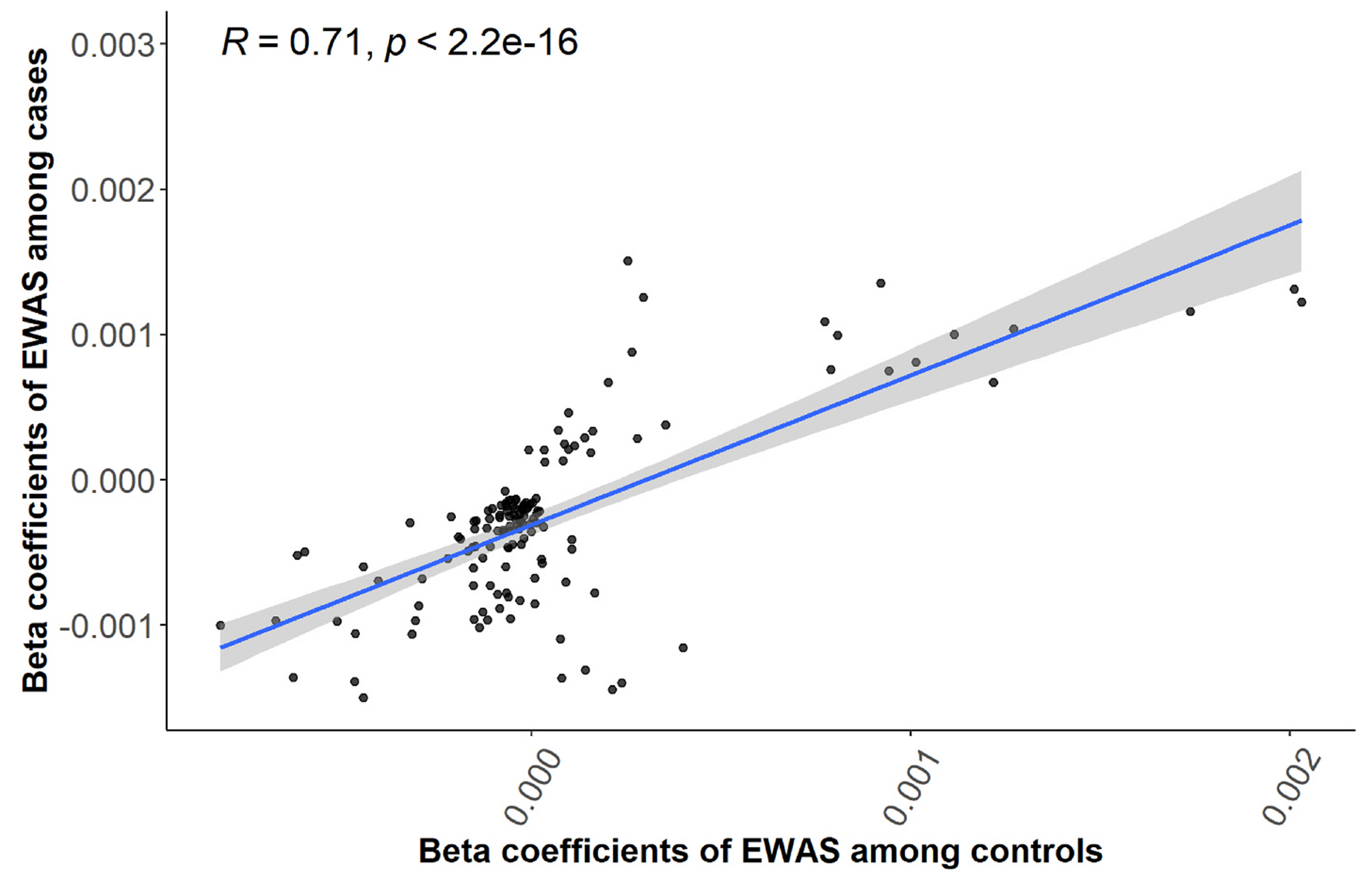
Correlation of beta coefficients (140 CpGs identified from the EWAS analysis were included) between PD cases and controls.

We identified 140 CpG sites as being associated with copper exposure at a B-H adjusted significance level (p < 10E-07) in the total study population (Supplement Table 1, Fig. 1). The top hit was cg17591573 (Chr6, pos = 33156206, p = 8.84E-09) in the *COL11A2* gene, which was negatively associated with copper exposure (r = −0.16). In analyses stratified by PD status, this CpG site had the same direction with exposure among PD patients (r = −0.16, p = 7.66E-09) and controls (r = −0.03, p = 0.77). Additional top CpG sites identified as differentially methylated include cg11538176 in *UBE3C* (r = −0.18, p = 9.26E-09), cg11208322 in *MAGI2* (r = −0.15, p = 1.00E-08), cg25788729 in *PLCD1* (r =−0.20, p =1.45E-08), cg11910759 (r = −0.20, p = 2.45E-08). Among the 140 significant CpGs identified in the total study population, 81 were also detected in PD patients (p < 10E-07), whereas only one CpG site (cg12986327 in the PTH2 gene) reached genome-wide significance in the non-PD control group. Although many CpG sites among these 140 did not reach the statistical significance threshold with copper exposure in controls (likely due to the much smaller sample size), most of the correlation coefficients were in the same direction as those observed in the total population (Pearson correlation R = 0.71, p = 2.2E-16; Fig. 2).

Among the EWAS results, the genomic inflation factor was 1.41 in the total study sample, driven largely by PD cases (*λ* = 1.35) with deflation in controls (*λ* = 4.50E-09), suggesting that signals are greatly case-driven while controls showed minimal variation. As a sensitivity analysis, we further applied the *bacon* R package (van Iterson et al., 2017), which uses a Bayesian approach based on an empirical null distribution, to correct for both inflation and bias in the t-statistics derived from the empirical Bayes regressions. After correction, the inflation factors were reduced to 1.24 for the total study sample, 1.21 for PD cases, and 0.93 for controls, indicating improved calibration of the test statistics. As expected, this correction also attenuated the overall p-values, and none of the CpG sites passed the original significance threshold of 10E-07 in the total study sample and PD cases. However, in non-PD controls, cg12986327 in the PTH2 gene (originally detected in EWAS) remained after correction (Supplement Table 2). To support interpretation, quantile–quantile (QQ) plots comparing the corrected and uncorrected p-values for the total sample, PD cases, and controls were presented in Supplement Figs. 5–7.

Detailed summary statistics stratified by PD status for these 140 CpGs from the original EWAS and the Bayesian corrected method can be found in Supplement Table 2.

Region level analysis identified 40 differentially methylated regions with at least 5 CpG sites in each of them (Supplement Table 3), the top hit located in chromosome 1 (bp starts at 205818668 and ends at 205819609, p = 8.35E-05).

### 3.3. Gene set enrichment and pathway analysis

**Fig. 3.**
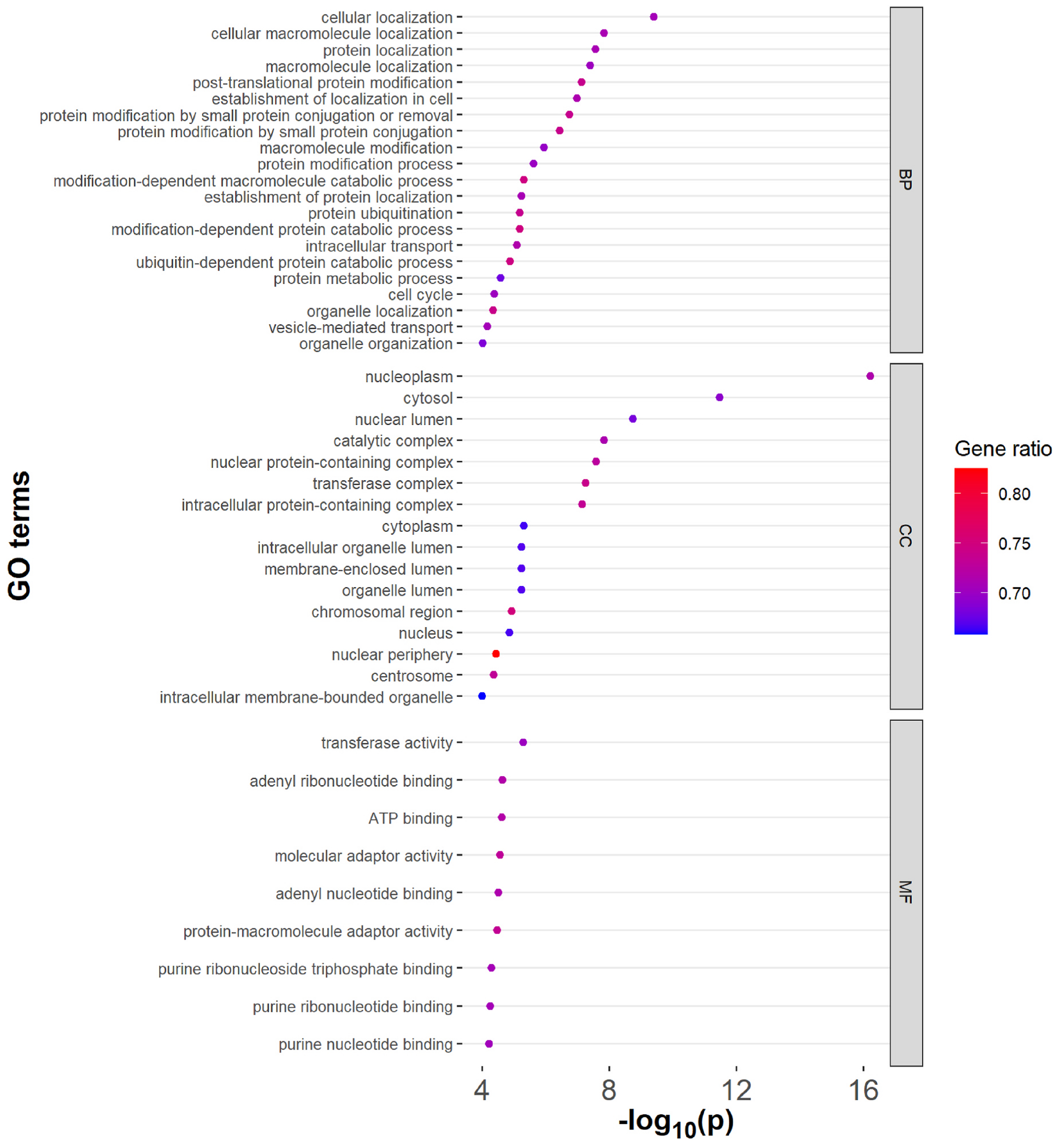
Overrepresented Gene Ontology (GO) terms in the total study sample (n = 806). Gene ratio stands for the percentage of differentially expressed genes in the given GO terms. Abbreviations: BP: biological process, CC: cellular component, MF: molecular functions.

At CpG level, GO analysis found a total of 46 GO terms to be overrepresented (FDR <0.05) in the entire study population (Fig. 3, Supplement Table 4). The top terms identified for cellular component, biological process, and molecular function were nucleoplasm (p = 5.94E-17, FDR = 1.33E-12), cellular localization (p = 3.91E-10, FDR = 2.92E-06), and transferase activity (p = 5.00E-06, FDR = 5.43E-03), respectively. For pathway analysis, we identified the overrepresentation of the following biological pathways: 1) Fc gamma R-mediated phagocytosis, 2) EGF receptor signaling, 3) integrin signaling, and 4) inflammation mediated by chemokine and cytokine signaling.

In the 40 genomic regions, the only GO term for biological processes we identified was homophilic cell adhesion via plasma membrane adhesion molecules at a p-value of 2.27E-07, FDR = 5.08E-03 (Supplement Table 4).

## 4. Discussion

EWAS results indicated that DNA blood methylation levels were associated with chronic copper exposure from agricultural pesticide use at 140 CpG sites and within 40 genomic regions among the 806 study participants (569 PD cases and 237 population controls). The top DMP and DMR were cg17591573 in the *COL11A2* gene and a region located in chromosome 1 with 9 CpG sites clustered together (bp starts from 205818668 and ends at 205819609). In addition, gene overrepresentation analysis identified multiple pathways potentially related to toxicity of copper pesticides, specifically phagocytosis, EGF receptor signaling pathway, and homophilic cell adhesion mechanisms.

In our previous EWAS of OP pesticide exposure with a smaller sample size with data from the first study wave only, we identified 70 CpG sites associated with OP exposure in blood sample (Paul et al., 2018). In the present analysis focused on copper pesticide exposure, none of those previously identified OP-related CpGs overlapped with the 140 CpGs associated with copper. This lack of overlap suggests distinct epigenetic signatures for these two pesticide classes, despite their moderate to strong correlation. These findings support our approach to adjust for OP exposure in the copper EWAS models to reduce confounding and reinforce the biological specificity of copper-associated methylation changes.

Among the 140 differentially methylated CpG sites, cg17591573 (r = −0.16, p = 8.84E-09) in the *COL11A2* gene exhibited the strongest association with copper exposure. *COL11A2* is an important protein coding gene for producing a component of a minor fibrillar collagen, which is widely believed to be associated with hearing impairment (Acke et al., 2012; De Leenheer et al., 2001). While this site was statistically significantly different with exposure, there is limited evidence linking *COL11A2* directly to PD or neuronal function. Notably, this gene is localized to the *HLA-DPB2* locus which lies within the major histocompatibility complex (MHC) region (Hanson et al., 1989). This raises the possibility of a connection between copper exposure and immune response contributing to PD pathogenesis, as the human leukocyte antigen (HLA) locus is known to be associated with PD and plays a critical role in its development (Yu et al., 2021).

Interestingly, cg11538176 in *UBE3C* (r = −0.18, p = 9.26E-09) and cg11208322 in *MAGI2* (r = −0.15, p = 1.00E-08) were also highlighted in the entire study population and were among the top hits in PD cases in stratified analyses. A previous in-vitro study found that the loss of *UBE3C* impairs the proteasome’s ability to fully degrade substrates, leading to the accumulation of protein fragments that may play a critical role in PD development (Chu et al., 2013). In terms of *MAGI2*, it acts as a scaffold molecule at synaptic junctions, coordinating the assembly of neurotransmitter receptors and cell adhesion proteins (The UniProt Consortium, 2025). A previous study suggested that a long non-coding RNA, MAGI2-AS3, which serves as an antisense transcript to the *MAGI2* gene, promotes a pro-inflammatory response in Alzheimer’s Disease (AD) and thus may be also of relevance to PD pathology (Liu et al., 2022). This gene was also detected in a GWAS study of PD and may affect PD etiology through positive regulation of neuron projection development (Mortezaei et al., 2017).

Aside from individual CpGs or genes, we observed that nucleoplasm (p = 5.94E-17, FDR = 1.33E-12), cellular localization (p = 3.91E-10, FDR = 2.92E-06), and transferase activity (p = 5.00E-06, FDR = 5.43E-03) were overrepresented in the copper associated gene sets. This is not surprising since copper pesticides were designed to be toxic to pathogens through interacting with nucleic acids and disrupting key enzyme activities in cells (Kiaune and Singhasemanon, 2011; McCallan, 1949). Additionally, enrichment analysis identified a phagocytosis pathway, aligning with previous study findings that copper exposure can inhibit phagocytosis and impair the clearance of dead/dying neurons, synapses or other unwanted materials by microglial and astrocytic cells and eventually lead to neuroinflammation and neurodegeneration. This suggests that copper pesticide exposure may play a critical role in modulating immune responses in the brain (Kitazawa et al., 2016; Tremblay et al., 2019).

Although not formally reaching statistical significance, we identified several potentially relevant pathways (i.e., EGF (epidermal growth factor) receptor signaling pathway, integrin signaling pathway, and inflammation mediated by chemokine and cytokine signaling pathways) associated with copper pesticide exposure using the *PANTHER* classification system. Previous research has reported that lower EGF levels are associated with cognitive decline, as measured by changes in MoCA scores, in a cohort study of 236 PD subjects followed for up to five years (*β* = 0.207, p = 0.014, per standard deviation of EGF increase from the mean) (Lim et al., 2016). The EGF receptor was also found to be associated with phagocytosis impairment by modulating macrophage activation and function possibly related to inflammation (Hardbower et al., 2016; Luo et al., 2024). Additionally, copper exposure has been shown to increase the release of pro-inflammatory cytokines and downregulate the expression of low-density lipoprotein receptor-related protein-1 (LRP1), a key regulator of *α*-synuclein neuronal uptake and mediates its spread within the brain, suggesting its potential contribution to the pathology of PD (Chen et al., 2022; Kitazawa et al., 2016).

In our region-level analysis, we also identified overrepresentation of homophilic cell adhesion via plasma membrane adhesion molecules (p = 2.27E-07, FDR = 5.08E-03), a finding consistent with a recent bioinformatics study that reported enrichment for this pathway in 31 substantia nigra tissue samples from PD patients that were compared to 17 substantia nigra samples from healthy controls (Zhou et al., 2023).

Our findings are further supported by a previous copper-focused EWAS conducted in non-neurological populations. For example, 16 CpG sites were associated with plasma copper levels across five independent Chinese population-based cohorts and linked several of these loci to genes involved in inflammatory regulation, including SBNO2 and BCL3 (Long et al., 2021). These genes are known to influence NF-*κ*B signaling and inflammatory cascades (El Kasmi et al., 2007; Puccio et al., 2018). Notably, methylation at BCL3 was found to be associated with serum C-reactive protein (CRP) levels, a key marker of systemic inflammation (Koenig et al., 1999).

However, two small studies (brain tissue from 9 PD dementia) cases and 9 age-matched controls have reported decreased copper levels in various brain regions affected by PD dementia or AD dementia (Scholefield et al., 2021, 2024). The authors argued that a reduction in copper might impair the activity of superoxide dismutase 1 (SOD1) and other essential enzymes, preventing the removal of harmful superoxide ions and hydrogen peroxide species from cells.

A meta-analysis of 18 studies of postmortem brains (211 PD cases and 215 controls) also reported that copper levels in the substantia nigra were lower (d, −2.00; 95 % CI, −2.81 to −1.19; P < 0.001) in PD cases (Genoud et al., 2020). This could primarily be attributed to the loss of copper binding to proteins such as ceruloplasmin, which may result in the release of free copper and eventually cause damage through oxidative stress and neurodegeneration (Ajsuvakova et al., 2020; Montes et al., 2014). Conversely, elevated copper levels in blood (serum or plasma) have shown associations with cognitive dysfunction in other studies (Li et al., 2023a; Mateo et al., 2023).

To date, this is the first study to examine the association between agricultural copper pesticide exposure and DNA methylation in a large, population-based case-control study. We included a large number (n = 569) of relatively early-stage PD cases and 237 community controls and further adjusted for OP exposure to increase the statistical power and account for potential confounding induced by OP. This adjustment was reflected in our pathway analysis, where the nicotinic acetylcholine receptor signaling pathway ranked among the least enriched. This finding contrasts with our previous work on differential methylation associated with OP exposure, where it was identified as the most enriched pathway (Paul et al., 2018). In addition, recall bias was minimized due to the record-based CAPUR data for copper pesticide exposure estimation.

However, several limitations should be acknowledged. One key limitation is the presence of genomic inflation in the test statistics, which suggests that residual confounding may persist despite adjustment for known covariates. Additionally, inflation in EWAS can result from correlation between neighboring CpG sites (i.e., CpG linkage disequilibrium) or from overestimation when a large number of smalleffect true associations are present across the genome (van den Berg et al., 2019). These factors can complicate interpretation and potentially reduce the precision of effect estimates. In addition, our blood samples were collected at a single time point. There is a potential for methylation levels to fluctuate and respond dynamically to environmental exposures. Thus, our data offered only a cross-sectional snapshot of DNA methylation in relation to chronic copper pesticide exposure and may not fully reflect the complexity of these epigenetic changes. Future analysis will aim to incorporate PD progression markers and possibly investigate additional molecular layers (e.g., metabolome, microbiome, etc.).

## 5. Conclusions

Our study provides new evidence that long-term copper pesticides exposure is associated with differential DNA methylation in blood of PD patients and elderly controls suggesting that chronic copper pesticide exposure may alter multiple pathways (e.g., phagocytosis pathway, EGF receptor signaling pathway, homophilic cell adhesion mechanisms) that are related to neuroinflammation and neurodegeneration in PD development.

## Supplementary Material

1

2

3

4

5

6

7

8

9

10

11

## Figures and Tables

**Table 1:** Demographic and exposure characteristics of PEG participants with blood derived methylation data (N = 806).

Characteristics	Overall, N = 806^a^	With PD, N = 569^a^	Without PD, N = 237^a^
**Age at blood draw (years)**	69.6 (10.9)	70.5 (9.8)	67.4 (12.8)
**Sex**			
Male	482 (59.8%)	356 (62.6%)	126 (53.2%)
Female	324 (40.2%)	213 (37.4%)	111 (46.8%)
**Smoking status**			
Non-smoker	397 (49.3%)	301 (52.9%)	96 (40.5%)
Former smoker	366 (45.4%)	242 (42.5%)	124 (52.3%)
Current smoker	43 (5.3%)	26 (4.6%)	17 (7.2%)
**Ethnicity**			
European ancestry	695 (86.2%)	468 (82.2%)	227 (95.8%)
Hispanic	93 (11.5%)	84 (14.8%)	9 (3.8%)
Other	18 (2.2%)	17 (3.0%)	1 (0.4%)
**Mean methylation**	0.3 (0.0)	0.3 (0.0)	0.3 (0.0)
**Copper count** ^b,c^	3.5 (3.9)	3.9 (4.1)	2.8 (3.3)
**OP count** ^b,d^	16.9 (13.4)	18.2 (13.8)	13.6 (11.7)

a Mean (SD); n (%).

b **Total number of chemicals exposed**: calculated by assigning a value of 1 if the yearly average poundage applied exceeded the median level observed in healthy controls, and 0 if not.

c **Copper chemicals (CADPR chem code) included**: copper carbonate (60), copper hydroxide (151), copper salts of fatty and rosin acids (155), copper oxychloride (156), copper oxychloride sulfate (158), copper sulfate (pentahydrate) (161), copper sulfate (basic) (162), copper-zinc sulfate complex (164), copper oxide (ous) (175), copper (714), copper dihydrazinium sulfate (753), copper triethanolamine complex (1615), copper sulfate, monohydrate (1789), copper ammonium complex (3550), and copper ethanolamine complexes (mixed) (3551).

d **OP chemicals (CADPR chem code) included**: monocrotophos (52), bensulide (70), dicrotophos (72), trichlorfon (88), carbophenothion (110), fensulfothion (181), ddvp (187), s,s,s-tributyl phosphorotrithioate (190), Dioxathion (192), diazinon (198), dimethoate (216), disulfoton (230), chlorpyrifos (253), fonofos (254), Ethion (268), merphos (293), azinphos-methyl (314), phosmet (335), malathion (367), oxydemeton-methyl (382), methyl parathion (394), ethoprop (404), naled (418), parathion (459), phorate (478), phosalone (479), mevinphos (480), phosphamidon (482), sulfotep (558), demeton (566), tepp (577), ethephon (1626), leptophos (1676), acephate (1685), methidathion (1689), methamidophos (1697), dialifor (1799), fenamiphos (1857), sulprofos (2006), profenofos (2042).

## Data Availability

The methylation data analyzed is available in the GEO repository, accession numbers GSE72774 and GSE72776.

## Appendix A. Supplementary data

Supplementary data to this article can be found online at https://doi.org/10.1016/j.envres.2025.122335.
