# Supplementary material for: Agricultural copper pesticide exposure and DNA methylation in Central Valley of California residents with and without Parkinson’s disease
Source: Environ Res. Author manuscript; Available in PMC 2026 May 11. (PMC13159479; doi:10.1016/j.envres.2025.122335)
Supplement: 1 [file NIHMS2169646-supplement-1.docx]

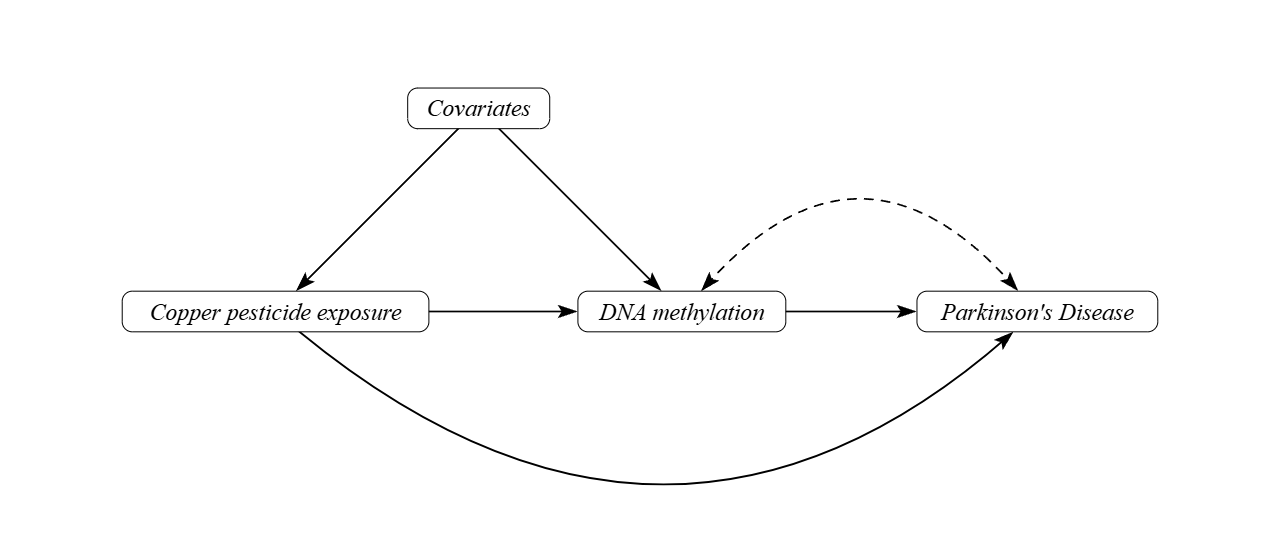


**Supplement Figure 1.** Directed acyclic graph (DAG) illustrating hypothesized relationships between copper pesticide exposure, DNA methylation, and Parkinson’s disease (PD). Copper exposure may influence PD risk both directly and indirectly via DNA methylation changes. Dashed arrow indicates the hypothesis that DNA methylation can act as both a mediator and a consequence of PD in this relationship.
