# Supplementary material for: Agricultural copper pesticide exposure and DNA methylation in Central Valley of California residents with and without Parkinson’s disease
Source: Environ Res. Author manuscript; Available in PMC 2026 May 11. (PMC13159479; doi:10.1016/j.envres.2025.122335)
Supplement: 5 [file NIHMS2169646-supplement-5.docx]

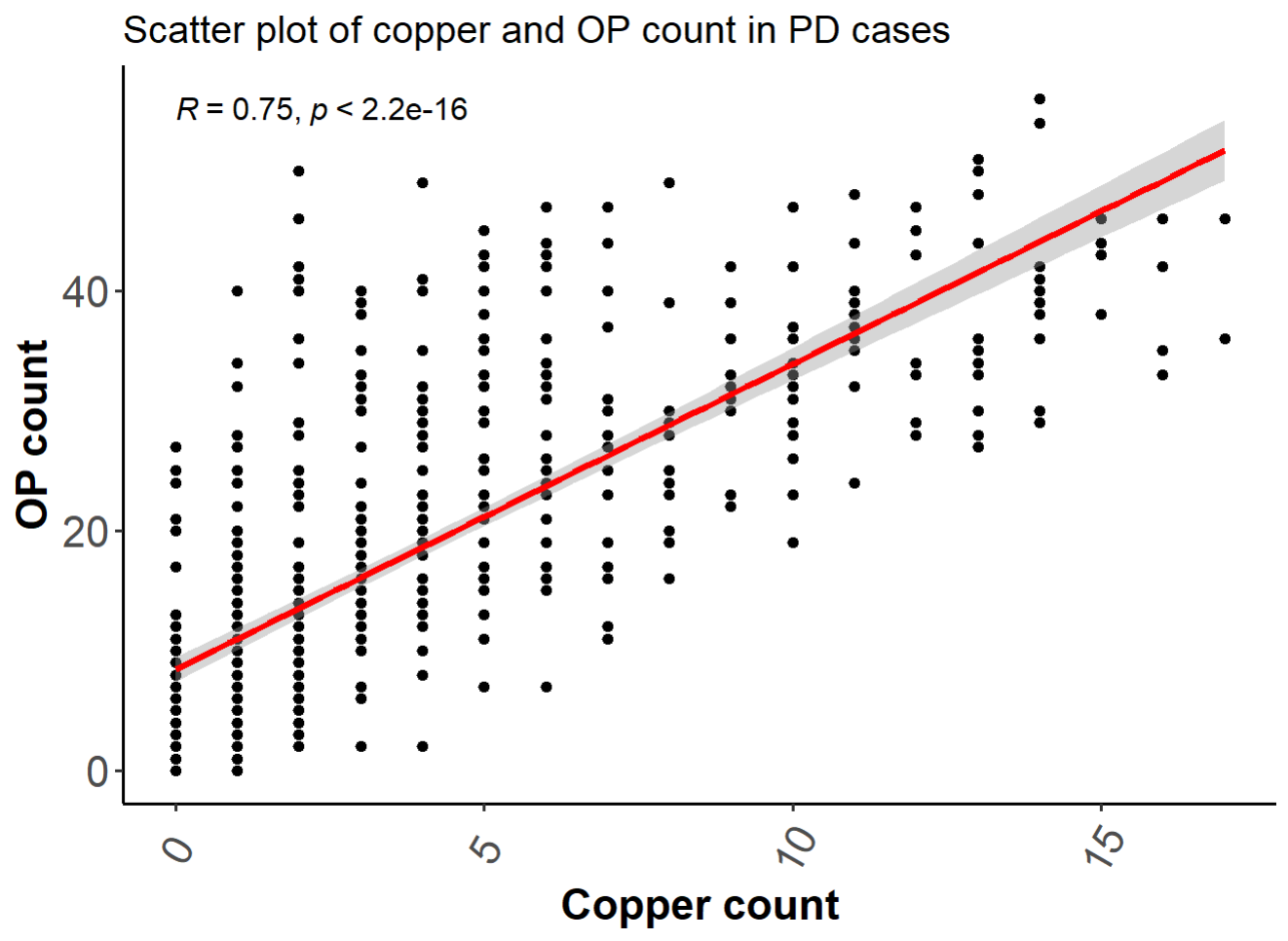


**Supplement Figure 3.** Scatter plot showing the relationship between copper pesticide count and organophosphate (OP) pesticide count in PD cases (n = 569).
