# Supplementary material for: Agricultural copper pesticide exposure and DNA methylation in Central Valley of California residents with and without Parkinson’s disease
Source: Environ Res. Author manuscript; Available in PMC 2026 May 11. (PMC13159479; doi:10.1016/j.envres.2025.122335)
Supplement: 7 [file NIHMS2169646-supplement-7.docx]

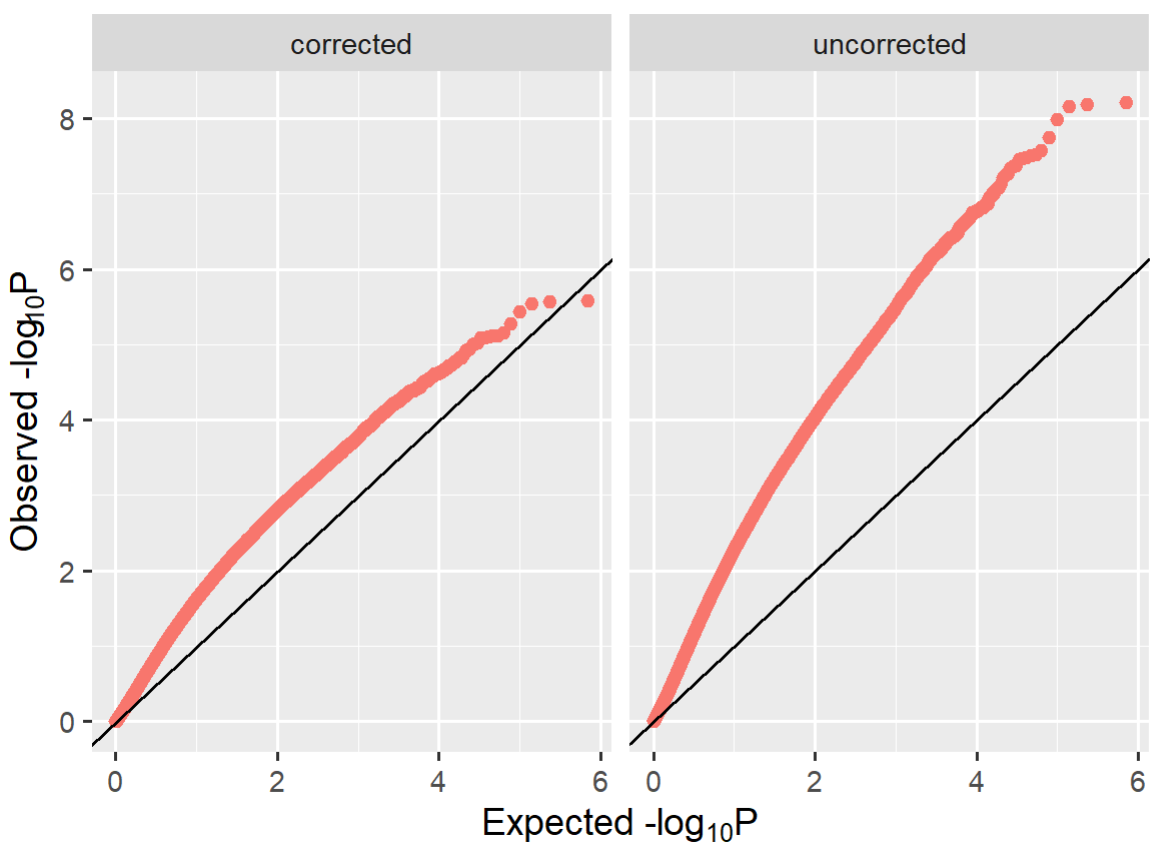


**Supplement Figure 5.** Quantile-quantile (QQ) plot of -log10 transformed P-values in the total study sample (n = 806)
