# Supplementary figures and images for: Agricultural copper pesticide exposure and DNA methylation in Central Valley of California residents with and without Parkinson’s disease
Source: Environ Res. Author manuscript; Available in PMC 2026 May 11. (PMC13159479; doi:10.1016/j.envres.2025.122335)

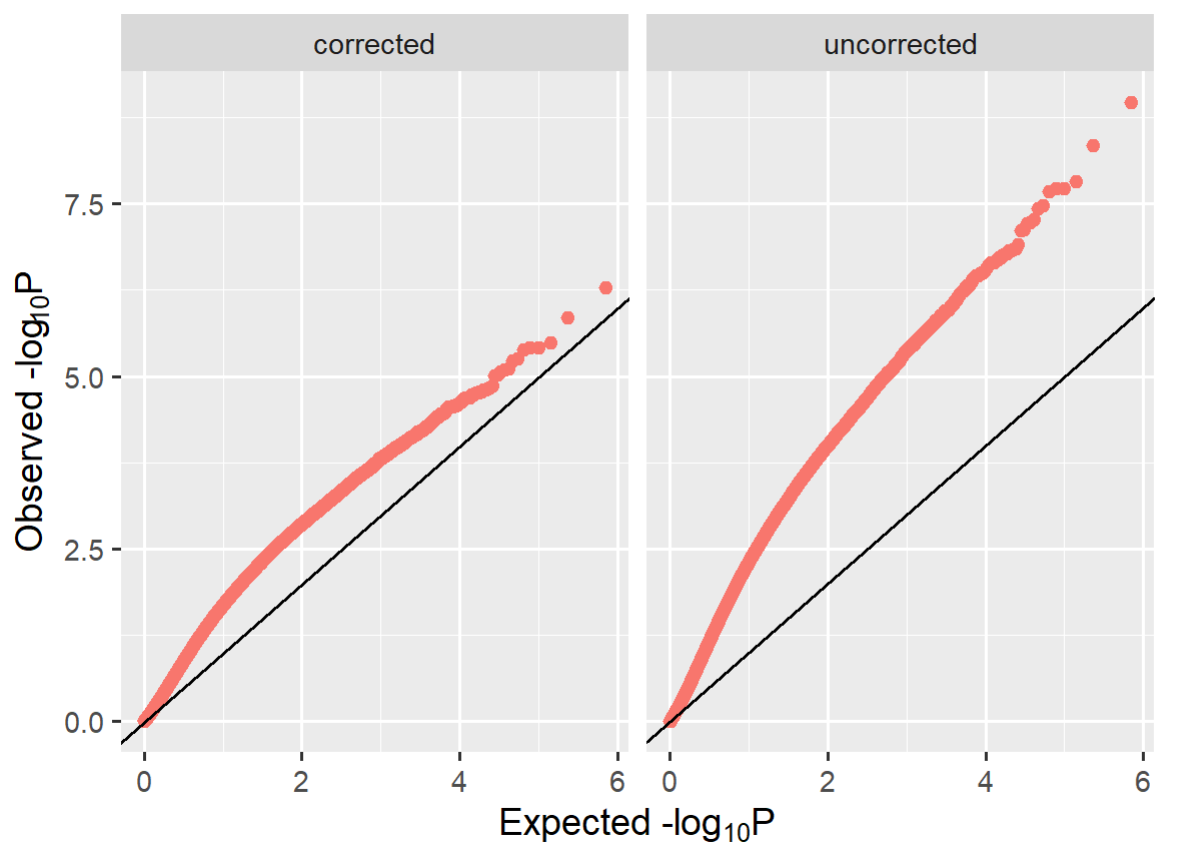


**Supplement Figure 6.** Quantile-quantile (QQ) plot of -log10 transformed P-values in PD cases (n = 569)

Supplement: 8 [file NIHMS2169646-supplement-8.docx]

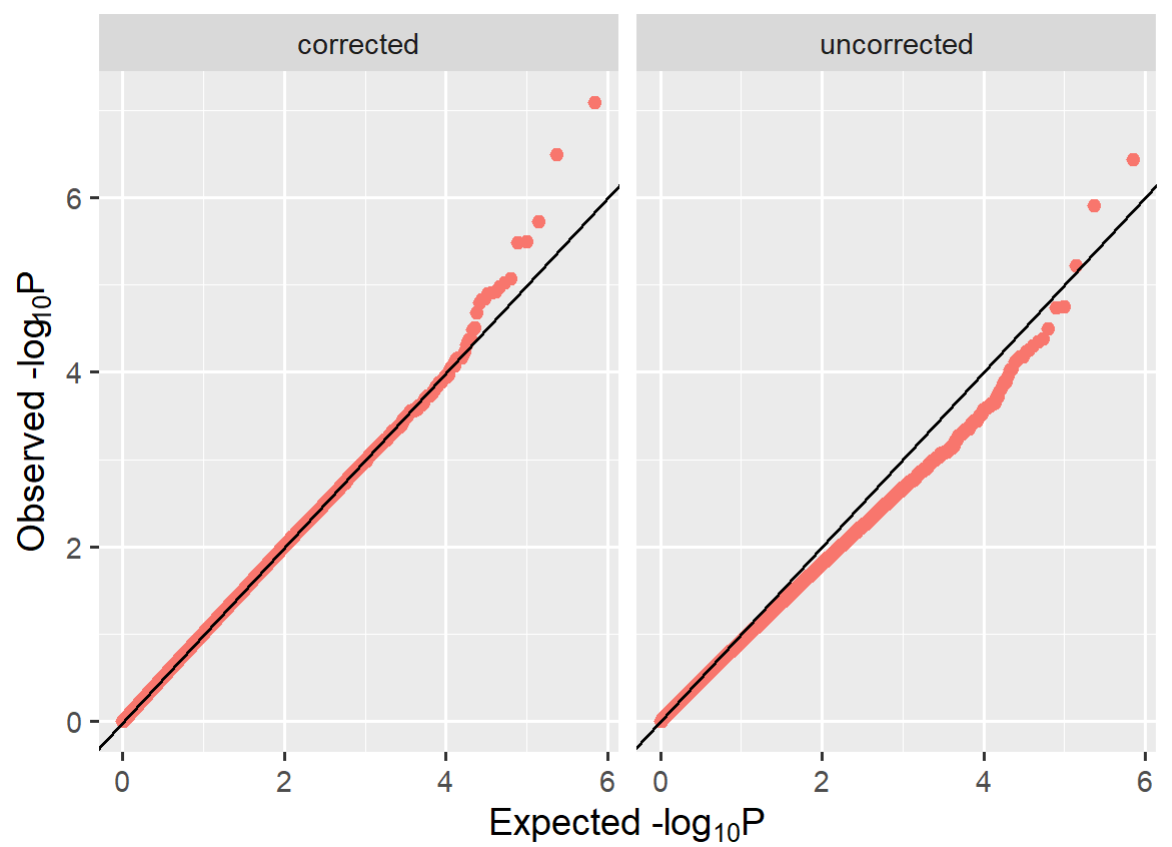


**Supplement Figure 7.** Quantile-quantile (QQ) plot of -log10 transformed P-values in non-PD controls (n = 227)

Supplement: 9 [file NIHMS2169646-supplement-9.docx]
